# Heterologous Prime-Boost Regimens Using rAd35 and rMVA Vectors Elicit Stronger Cellular Immune Responses to HIV Proteins Than Homologous Regimens

**DOI:** 10.1371/journal.pone.0045840

**Published:** 2012-09-26

**Authors:** Silvia Ratto-Kim, Jeffrey R. Currier, Josephine H. Cox, Jean-Louis Excler, Anais Valencia-Micolta, Doris Thelian, Vicky Lo, Eddy Sayeed, Victoria R. Polonis, Patricia L. Earl, Bernard Moss, Merlin L. Robb, Nelson L. Michael, Jerome H. Kim, Mary A. Marovich

**Affiliations:** 1 United States Military HIV Research Program, Walter Reed Army Institute of Research, Rockville, Maryland, United States of America; 2 International AIDS Vaccine Initiative, New York, New York, United States of America; 3 Laboratory of Viral Diseases, National Institute of Allergy and Infectious Diseases, National Institutes of Health, Bethesda, Maryland, United States of America; National Institute of Allergy and Infectious Diseases, United States of America

## Abstract

We characterized prime-boost vaccine regimens using heterologous and homologous vector and gene inserts. Heterologous regimens offer a promising approach that focuses the cell-mediated immune response on the insert and away from vector-dominated responses. Ad35-GRIN/ENV (Ad35-GE) vaccine is comprised of two vectors containing sequences from HIV-1 subtype A *gag*, *rt*, *int*, *nef* (Ad35-GRIN) and *env* (Ad35-ENV). MVA-CMDR (MVA-C), MVA-KEA (MVA-K) and MVA-TZC (MVA-T) vaccines contain *gag*, *env* and *pol* genes from HIV-1 subtypes CRF01_AE, A and C, respectively. Balb/c mice were immunized with different heterologous and homologous vector and insert prime-boost combinations. HIV and vector-specific immune responses were quantified post-boost vaccination. Gag-specific IFN-γ ELISPOT, intracellular cytokine staining (ICS) (CD107a, IFN-γ, TNF-α and IL-2), pentamer staining and T-cell phenotyping were used to differentiate responses to inserts and vectors. Ad35-GE prime followed by boost with any of the recombinant MVA constructs (rMVA) induced CD8+ Gag-specific responses superior to Ad35-GE-Ad35-GE or rMVA-rMVA prime-boost combinations. Notably, there was a shift toward insert-focus responses using heterologous vector prime-boost regimens. Gag-specific central and effector memory T cells were generated more rapidly and in greater numbers in the heterologous compared to the homologous prime-boost regimens. These results suggest that heterologous prime-boost vaccination regimens enhance immunity by increasing the magnitude, onset and multifunctionality of the insert-specific cell-mediated immune response compared to homologous vaccination regimens. This study supports the rationale for testing heterologous prime-boost regimens in humans.

## Introduction

The development of an efficacious preventive HIV-1 vaccine was given a significant impetus following the promising results of the RV144 Thai trial [1]. Modest efficacy against HIV acquisition was conferred by a heterologous prime-boost regimen using a canarypox vector ALVAC-HIV (vCP1521) expressing *env*, *gag* and *pol* genes and AIDSVAX B/E, a bivalent gp120 envelope glycoprotein. This regimen elicited modest interferon-gamma (IFN-γ) ELISPOT responses but strong CD4+ lymphoproliferative and humoral immune responses that were better than either product tested alone. In contrast, the vaccine regimen used in the STEP study was a homologous recombinant Adenovirus (rAd) vaccine regimen given in three doses (MRKAd5 *gag*, *pol*, *nef* HIV vaccine), which despite robust CD8+ T cell IFN-γ ELISPOT responses to both inserts and vector, resulted in no protection from infection and no decrease in plasma HIV viral load [2].

The heterologous prime-boost strategy is widely used in the design of vaccines regimens against other infectious diseases, and in cancer immuno-therapy [3], [4], [5], [6], [7], [8]. Pre-clinical and clinical studies using heterologous prime-boost HIV-1 vaccines have shown the generation of stronger and broader immune responses [9], [10], [11]. In practice, the heterologous prime-boost strategy may apply to either different vectors, different insert sequences, or as tested in this study, a combination of different vectors and different inserts. Such strategies have several important theoretical advantages: (1) a shift in the immunodominance hierarchy by focusing the immune response on the inserts rather than the vectors; (2) an increase in the breadth and depth of the immune response due to priming and boosting with different insert sequences; and (3) generation of unique phenotypic and functional signatures of the immune response by virtue of the unique innate immune response induced by each vector. The heterologous vector approach may also avoid the consequences of pre-existing anti-vector immunity. DNA is commonly used as a prime since it is easy to manufacture and pre-existing anti-vector immunity is not an issue [7], [10]. Recently, HIV-1 vaccine development has focused on low serotype prevalence rAd as potential vaccine vectors [12], [13], [14] and attenuated pox virus vectors [15]. Combinations of rAd and recombinant MVA (rMVA) have been tested in pre-clinical and some clinical settings with mixed results [16].

We tested two rAd35 and three rMVA constructs to better understand and evaluate immune responses elicited after homologous versus heterologous prime boost regimens. The goal of this study was to evaluate the contribution of the vector prime boost regimen on the development of the cellular immune responses to the HIV gene inserts. IFN-γ ELISPOT, multiparameter flow cytometry and MHC Class I - pentamer-binding assays were used to measure insert and vector specific responses. The heterologous prime-boost regimen gave rise to potent, cross-reactive, multifunctional effector memory T cells that focused the response on the HIV-1 inserts rather than the vectors.

## Materials and Methods

### Mice and vaccination protocol

**Figure 1:**
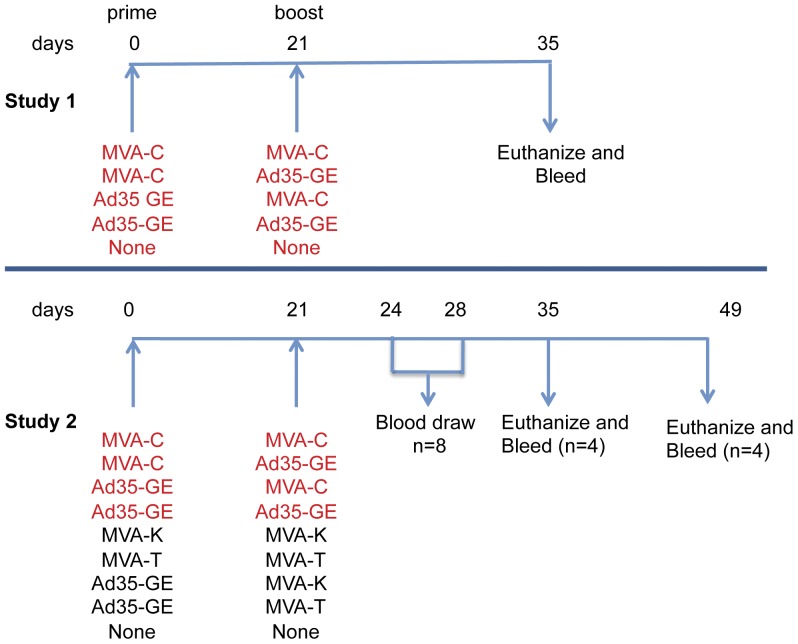
Schematic of the two studies. Study 1 tested 4 vaccine combinations (5 mice/group) plus 10 naïve mice. Spleens and serum were collected at day 14 after the boost corresponding to day 35 on the schematic. Study 2 included the set of pairings from the first study (highlighted in red) in addition to the pairings indicated. Each group included 8 BALB/c mice that were vaccinated at days 0 and 21 as indicated. In this study, serum and blood were also harvested at the time indicated in the schematic. rMVA and rAd35 constructs were injected intramuscularly at 1×10^7^ pfu and 2×10^10^ vp, respectively.

Two separate animal studies were conducted and analyzed to meet our objective (Figure 1). Female BALB/c mice (5 mice per group in the first study and 8 mice per group in the second study) were purchased from Hilltop Lab Animals, Scottdale, PA and housed at BIOCON Inc. (Rockville, MD). All vaccinations, bleeds and spleen removals were conducted at BIOCON. Spleens and blood were transported to the immunology laboratory within 1 hour of collection. The study was reviewed and approved by the BIOCON Institutional Animal Care and Use Committee and the U.S. Army Medical Materiel Development Activity, Ft. Detrick, MD. Research was conducted in compliance with the Animal Welfare Act and other federal statutes and regulations relating to animals and experiments involving animals and adheres to principals stated in the *Guide for the Care and Use of Laboratory Animals*, NRC Publication, 1996 edition.

In Study 1, MVA-CMDR (MVA-C) was used in combination with the Ad35-GRIN/ENV (Ad35-GE) to assess whether heterologous vector prime-boost vaccination elicited different immunological responses than homologous vaccination. Study 2 was performed to confirm Study 1 and to study the other MVA constructs [MVA-KEA (MVA-K) and MVA-TZC (MVA-T)] in various combinations with the Ad35-GE to assess the respective contributions of the HIV inserts and vectors to induced immune responses.

### Vaccines

Two Ad35 vectors were used: Ad35-GRIN expressing HIV-1 subtype A *gag*, *RT*, *integrase*, *nef* genes (GRIN) as a fusion protein and Ad35-ENV expressing HIV-1 subtype A gp140 *env* gene (Transgene SA Illkirch-Graffenstaden, France). Ad35-GRIN and Ad35-ENV were co-formulated (Ad35-GE) in the same vial and injected by the intramuscular route (IM) at 2×10^10^ viral particles (vp) per mouse (1×10^10^ vp for each construct) (Table 1).

**Table 1:** Vaccine Constructs Used to Immunize the Mice and Matching Reagents.

Vectors	HIV gene inserts	Subtype	Gag epitope variant motif	Gag Insert Matching peptides	Env Insert Matching peptides
Ad35-GE	*gag, reverse transcriptase, integrase, nef, env*	Subtype A	KDTI	P1 Gag, Gag A VIP	P1 Env
MVA-C	*gag*, *env* and *pol*	Subtype CRF01_AE	KETI	CMDR Gag, Gag A VIP	CMDR Env
MVA-T	*gag*, *env* and *pol*	Subtype C	KDTI	P1 Gag, Gag A VIP	
MVA-K	*gag*, *env* and *pol*	Subtype A	KETI	CMDR Gag, Gag A VIP	

rMVA constructs were designed and constructed as described previously [17]. Each rMVA contains HIV-1 *gag*, *env* (gp150) and *pol* genes. MVA-CMDR carries CRF01_AE genes from Thailand (MVA-C), MVA-KEA contains subtype A genes from Kenya (MVA-K) and MVA-TZC contains subtype C genes from Tanzania (MVA-T). MVA constructs were injected by the IM route at 1×10^7^ plaque forming unit (pfu) per mouse (Table 1). The vaccination regimens are shown in Figure 1. Mice were bled via ocular puncture at days 24 and 28. Mice were euthanized and blood and spleens were collected on day 35 and day 49 from each group (Figure 1).

### Spleen processing

Individual spleens were homogenized to prepare single cell suspensions. ACK Lysing Buffer (Lonza, PA) was used to lyse red blood cells. After washing, cells were filtered through a 100 µM cell strainer and counted by Guava PCA system, Cytosoft 6.0.1 (Guava Technologies-Millipore Corp, Billerica, MA). Cells from the same group were pooled for the ICS, pentamer and phenotyping studies but were tested individually in the IFN-γ ELISPOT.

### Whole blood processing

Blood was collected by retro-orbital puncture according to IACUC approved procedures. Samples from the same group were pooled and diluted up to three times the original volume. The diluted blood was carefully layered on Ficoll-Paque and centrifuged at 400×g for 40 min. at 18–20°C. Mononuclear cells were harvested, washed and counted by Guava PCA system (Guava Technologies-Millipore Corp).

### Peptides and Antigens

The peptide sets matching the Gag (CM240) and Env (CM235) insert sequences in the MVA-C vaccine were manufactured by JPT Peptide Technologies Inc. (Berlin, Germany). The peptide sets spanning each protein consists of 95 and 138 individual peptides, respectively. Peptides are 15 to 18 amino acids in length overlapping by 10 to 12 amino acids. Each peptide set was pooled (by co-lyophilization at JPT Peptide Technologies Inc.) to make complete CMDR-Gag and CMDR-Env peptide pools, respectively. In the ELISPOT assays, a subtype A Gag Variant Inclusive Peptide (VIP) set was also used (JPT Peptide Technologies Inc.). This peptide set is composed of 207 individual peptides of 15 to 18 amino acids in length overlapping by 10 to 12 amino acids and was optimized for maximum coverage of the most frequent 10-mer epitope variants in the HIV subtype A epidemic (Currier J. et al, Journal of Translational Medicine). For the first set of experiments, Gag- and Env-specific peptides that matched the Ad35-GE were synthesized by AnaSpec (Fremont, CA). The peptides were 15mers with 11 amino acid overlap and pooled into 100–200 peptides depending on the protein. In addition, single Gag peptides were tested as they are known to be immunodominant in BALB/C mice. The peptides designated KETI (AMQMLK**E**TI) and KDTI (AMQMLK**D**TI) were synthesized in-house (U.S. Military HIV Research Program) with free amino termini using F-moc chemistry and standard solid-phase techniques using an ABI 433A Automated Peptide Synthesizer (Applied Biosystems, Foster City, CA). All peptides were >80% pure as determined by HPLC analysis on an Acquity Ultraperformance LC instrument (Waters Corp. Milford, MA), and verified for the correct sequence by N-terminal Edman degradation chemistry amino acid sequencing on an ABI Procise 490A peptide sequencer (Applied Biosystems). In addition to peptides specific for the inserts, a series of peptides were used to test the T cell response to the vectors. An overlapping peptide set (16×11 amino acids) matching the Ad5 hexon protein (HAdV5 Hexon) (synthesized by JPT Peptide Technologies Inc.) and the Ad35 hexon protein (courtesy of Dan Barouch, BIDMC, Boston, MA) were used in Study 1 and 2, respectively. To assess responses to the MVA construct, the minimal epitope H-2D^d^ restricted MVA050 (E3L) 140–148 (VGPSNSPTF), was synthesized and assessed for purity in-house as described above, at the U.S. Military HIV Research Program. This epitope is known to be immunodominant in BALB/C mice immunized with MVA vaccines [18].

### Intracellular cytokine staining of mouse splenocytes

Pooled mouse splenocytes (1.5×10^6^/mL) were stimulated for 6 hours at 37°C with either 1 µg/mL of the relevant peptides in duplicate wells or media alone. Anti-CD107a FITC antibody (BD Pharmingen, clone 1D4B), Brefeldin A and Monensin were added at the beginning of the stimulation. After washing, cells were incubated for 30 min. at room temperature (RT) with Aqua Live/Dead Stain. To block Fc receptors, CD16/32 antibody (BD Pharmingen, clone 2.4G2) was added to the cells. Surface staining was performed, and cells were incubated 30 min. at RT with anti-CD8 PerCP-Cy5.5 (BD Pharmingen, clone 53-6.7), and anti-CD19 PE-Cy5 (Proimmune, clone 6D5) antibodies. After fixation and permeabilization, cells were stained for 30 min. with anti-IFN-γ Pacific Blue (PB) (eBiosciences, clone XMG1.2), anti-interleukin 2 (IL-2) APC (BD Pharmingen, clone JES6-5H4), anti-tumor necrosis factor (TNF-α) PE-Cy7 (BD Pharmingen, clone MP6-XT22), and washed and resuspended in staining buffer containing sodium azide. Approximately 500,000 cells were acquired on a LSRII cytometer using BD FACSDiva software (BD Biosciences, San Jose, CA) and analyzed using FlowJo software (Tree Star, Cupertino, CA). Analyses and presentation of distributions were performed using SPICE version 5.1 [19]. Comparisons of distributions were performed using a Student's t test and a partial permutation test as previously described [19].

### MHC Pentamer Staining

Two million mouse splenocytes and/or PBMC were incubated in duplicate for 30 min. at RT with 100 µL of Aqua Live/Dead Stain. After washing, cells were incubated for 10 min. at RT with R-PE-labeled pentamer (ProImmune, Oxford, UK) bound to the MVA-050 immunodominant peptide, APC-labeled pentamer bound to the HIV Gag p24 peptide AMQMLKDTI, and/or R-PE-labeled pentamer bound to the HIV Gag p24 peptide AMQMLKETI. Cells were then incubated with FcR Block (CD16/32) and eFluor450 conjugated anti-CCR7 antibodies (eBioscience, clone 4B12) for 15 min. at 37°C. After washing, surface staining was performed, and cells were incubated 30 min. at RT with anti-CD8 PerCP-Cy5.5 (BD Pharmingen, clone 53-6.7), anti-CD62L APC-Cy7 (BD Pharmingen, clone MEL-14), anti-CD19 PE-Cy7 (BIOLEGEND, clone 6D5), and anti-CD127 PE-Cy5 (eBioscience, clone A7R34). Cells were then fixed with 2% formaldehyde for 15 min and acquired on a LSRII cytometer and analyzed as described in the previous section.

### IFN-γ ELISPOT Assay

IFN-γ ELISPOT assays were performed as described previously [17]. Briefly, 96-well nitrocellulose plates were pre-wet, washed and coated overnight with anti-mouse-IFN-γ mAb clone AN-18 (Mabtech, Cincinnati, OH) at 4°C. The plates were washed and blocked with 10% FBS-RPMI for a minimum of one hour. A total of 5×10^6^ splenocytes were plated in replicate of 5 with the relevant peptides (1 µg/mL/peptide) or media only and incubated for 18–24 hours. Production of IFN-γ by CD8+ T cells was detected by addition of biotinylated anti-IFN-γ mAb Clone R4-6A2 (Mabtech, Cincinnati, OH). ELISPOT development consisted of a one hour incubation at RT with avidin horseradish peroxidase complex (Vectastain® ABC kit, Vector Labs, Burlingame, CA) in PBS/0.05% Tween-20 buffer followed by washing with PBS, and incubation at RT with peroxidase substrate AEC for 4 min. (AEC substrate Kit, Vector Labs, Burlingame, CA). ELISPOT plates were evaluated with an automated ELISPOT reader system (Carl Zeiss, Thornwood, NY, and S5Core ImmunoSpot analyzer, Cellular Technology Ltd, Shaker Heights, OH). The results are expressed as the number of IFN-γ spot forming cells (SFC)/10^6^ spleen cells.

## Results

### Heterologous prime-boost regimens elicit stronger HIV immune responses than homologous regimens

Balb/c mice were immunized with different vaccination regimens and sampled as described in Figure 1. ICS assays were tested in both studies with identical reagents at 2 weeks post-boost (day 35), and for Study 2, ICS was also performed 4 weeks post-boost (day 49). The peak of the response was day 35 and the pattern or responses were similar between day 35 and day 49; hence the data reported focus on day 35 samples and combine the ICS responses from the two studies. IFN-γ ELISPOT assays were only tested in Study 1 and spleens were tested individually (n = 5).

**Figure 2:**
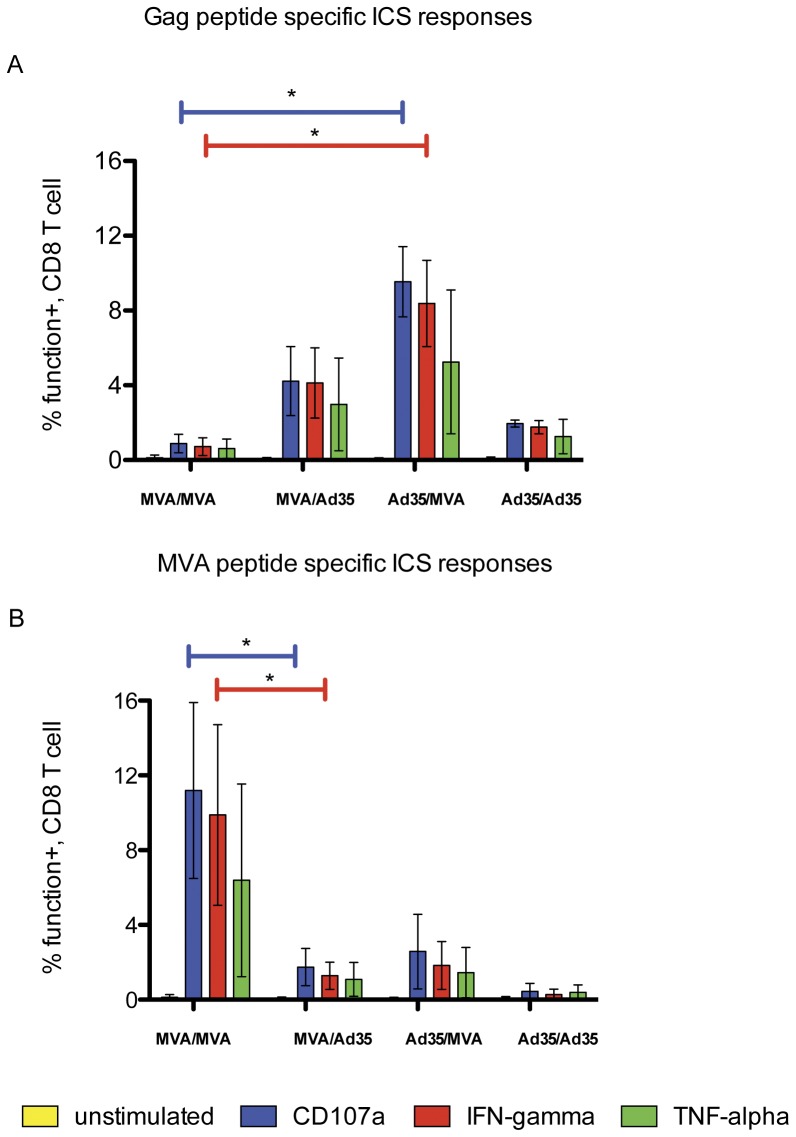
CD8+ ICS assays: CD8+ T-cell functional responses to the A) Gag immunodominant variant KDTI and B) the immunodominant 050 MVA peptide. The responses are shown as the mean plus Standard Deviation calculated by combining the results from both studies. Vaccination regimens are on the X-axis, MVA = MVA-C and Ad35 = Ad35-GE; horizontal color coded bars show which cytokine or degranulation marker reached a statistically significant increase (p<0.05). Kruskall-Wallis test and Dunn's Multiple Comparison test were applied. Only significant results are shown.

The ICS assays showed clearly that the heterologous prime-boost regimen induced higher CD8+ HIV-1 Gag responses than the homologous regimens (Figure 2A). In particular, splenocytes from Ad35-GE/MVA-C regimen mounted a statistically significant higher response (p<0.05) to Gag pools and to the immunodominant Gag epitopes, as measured by CD107 and IFN-γ expression when compared to splenocytes from homologous MVA-C/MVA-C regimen. In contrast, the homologous prime-boost MVA-C/MVA-C regimen focused the immunological responses on the MVA vector (Figure 2B). The response to the MV050 peptide was higher (p<0.05) when compared to the heterologous regimen MVA-CCMDR/Ad35-GE (Figure 2B, p<0.05). Similar vector responses were confirmed by IFN-γ ELISPOT (data not shown). The vaccine combinations tested in the second study (Figure 1) confirmed that the heterologous combination was superior for the magnitude of the response to the HIV-1 insert compared to the homologous combination (Table 2). Interestingly, among the homologous regimens, although the vector backbone was the same the responses to the immunodominant peptides and vector varied. For example, the MVA-T construct seemed to induce a lesser immunological response to the inserts while maintaing a similar response to the vector; conversely the MVA-K seemed to induce just the opposite. Also, despite the insert matching the KDTI peptide motif, the immune response induced by MVA-T homologous regimens was inferior to that induced by the mismatch contructs (Table 2). We may speculate that immunological responses are indeed influenced by the insert sequences and that some viral sequence can possibly be more immunogenic than others. Unfortunately, only the MVA reagents yielded a measurable response. It is possible that the Ad5 or Ad35 hexons peptide pools did not contain the correct epitope elicited by this vaccine or the responses were too low to be detected with the assays used.

**Table 2:** Percentage expression of cytokines or CD107 marker in CD8 splenocytes from the different vaccine groups in response to immunodominant inserts or vectors.

		KDTI			KETI			MVA050			Exon35		
		cytokine/marker											
vaccine groups	Peptide	IFN-γ	CD107	TNF-α	IFN-γ	CD107	TNF-α	IFN-γ	CD107	TNF-α	ΦN-γ	CD107	TNF-α
MVA-C-MVA-C	E/E	1.13*	1.3	1.1	2.7	2.8	0.8	14.1	15.3	10.9	0.0	0.0	0.2
MVA-K-MVA-K	E/E	2.3	2.9	1.8	4.0	4.8	3.1	4.7	6.3	3.4	0.0	0.1	0.3
MVA-T-MVA-T	D/D	1.0	1.1	1.2	0.6	0.8	1.0	9.9	11.4	8.1	0.0	0.1	0.6
Ad35-GE-MVA-T	D/D	4.3	4.7	3.9	2.0	2.6	1.9	2.6	3.4	2.5	0.3	0.3	0.6
Ad35-GE-Ad35-GE	D/D	2.1	2.1	2.1	1.2	1.1	1.2	0.5	0.8	0.7	0.1	0.2	0.4
Ad35-GE-MVA-C	D/E	10.3	11.1	8.6	8.5	9.4	6.6	2.9	4.3	2.6	0.0	0.1	0.2
Ad35-GE-MVA-K	D/E	8.0	8.9	7.0	6.9	7.6	6.0	3.8	5.3	3.8	0.0	0.0	0.3
naïve		0.0	0.1	0.2	0.0	0.1	0.2	0.0	0.1	0.2	0.0	0.0	0.2

* values represented are means of ICS experiments run in duplicate.

**Figure 3:**
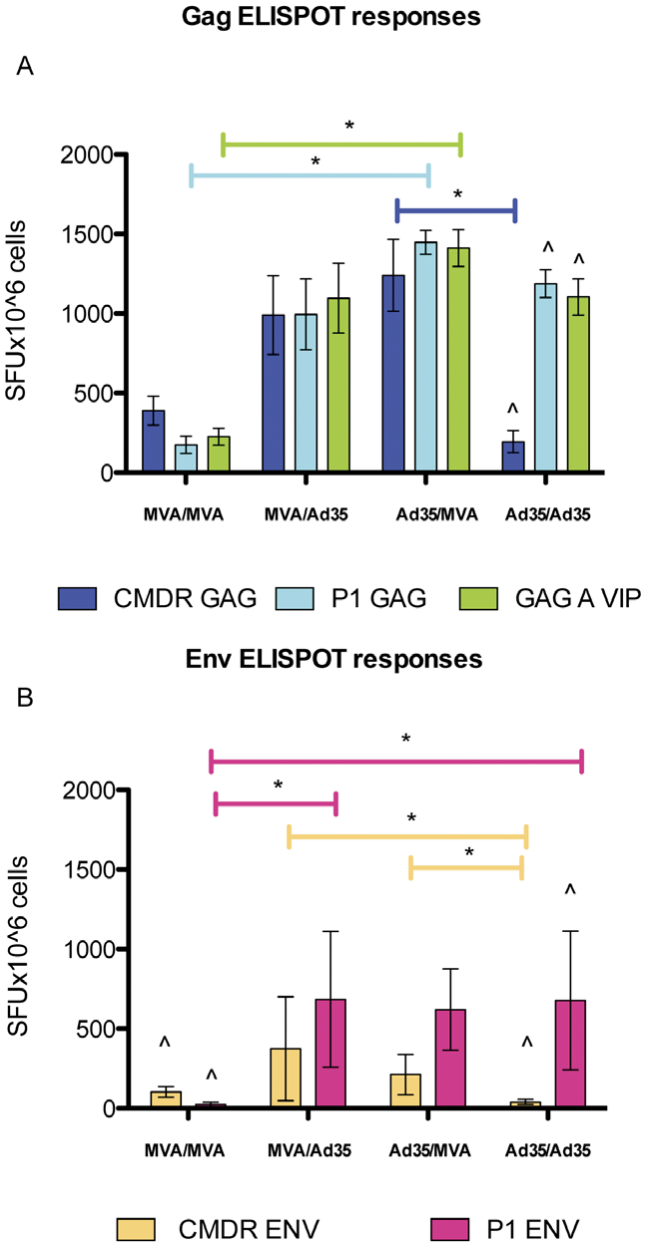
IFN-γ ELISPOT results from Study 1. Graphs represent means SFU/10^6^ cells + standard deviations of the responses to A) Gag peptide pools and B) envelope peptide pools. CMDR pools match the MVA-C vaccine inserts sequences, P1 peptide pools match the Ad35-GE inserts sequences. GAG A VIP is a peptide pool designed to maximize coverage of the most frequent 10-mer epitope variants in the HIV subtype A epidemic. Vaccination regimens are on the X-axis: MVA = MVA-C and Ad35 = Ad35-GE, horizontal bars show which peptide pool response reached a statistically significant increase. Kruskall-Wallis test and Dunn's Multiple Comparison test were applied. Only significant results are shown. ∧ symbol identify responses to peptide pools that were statistically significant different (p<0.05 Mann Whitney test) within the same regimen.

For the IFN-γ ELISPOT the responses to Gag pools that matched Ad35 (KDTI) and GAG A VIP (contains both) were significantly higher in the Ad35-GE/MVA-C regimen compared to the MVA-C homologous regimen (Figure 3A). A possible explanation is that the responses to the KDTI motif-containing peptide pools were not as well recognized in the MVA-C/MVA-C vaccinated animals (the CMDR Gag insert contains the KETI motif). The same observation was made when the homologous Ad35-GE regimen was compared to the heterologous Ad35-GE/MVA-C regimen (Figure 3A), the IFN-γ ELISPOT response to the CMDR Gag pool (contains the KETI motif) was low in splenocytes from mice immunized with the Ad35-GE homologous regimen while the responses to the homologous peptide pools were high, showing that Ad35-GE/Ad35-GE regimen induces splenocytes that have a less ability to cross-react to the different insert peptide pool. In addition, within the same regimen (Ad35-GE/Ad35-GE) there was a statistically significant difference (p<0.05) between the responses to the CMDR pools and the P1 Gag and Gag A VIP underscoring that the immunological response induced by this regimen is strong but very specific to the homologous sequence motif. Envelope responses were also measured by IFN-γ ELISPOT and showed a similar pattern to the Gag responses with the heterologous prime-boost regimens eliciting statistically significant higher responses (p<0.05) to both envelope pools and the homologous regimens restricting the response to their insert matched envelope pool (Figure 3B). In order to understand the respective contributions of the HIV inserts and vectors to the immune responses, we studied the combination of Ad35-GE with other rMVA vectors with inserts that partially match sequences to the Ad35 inserts. MVA-T (subtype C) contained the same KDTI motif as the Ad35-GE Gag insert, and MVA-K (subtype A) matched the subtype of the Ad35-GE but with the KETI motif in the Gag insert (Table 1). In addition, we studied the combination of the same vectors carrying different HIV genes (heterologous inserts) to evaluate if the immune responses could be amplified. By ICS assay with the three markers used (IFN-γ, CD107a and TNF-α), priming with Ad35-GE consistently elicited stronger immune responses with the three different rMVA boosts than with any other vector combinations (Table 2). The responses to the vectors remained consistently lower in the heterologous prime-boost compared to the homologous regimens, with MVA-C eliciting the strongest vector response (Table 2). These data are in agreement with those generated previously with the same constructs by Earl et al. [17] where anti-MVA vector responses were greatly boosted after the second vaccination with the homologous vector.

### Memory phenotype of pentamer positive CD8+ T cells

**Figure 4:**
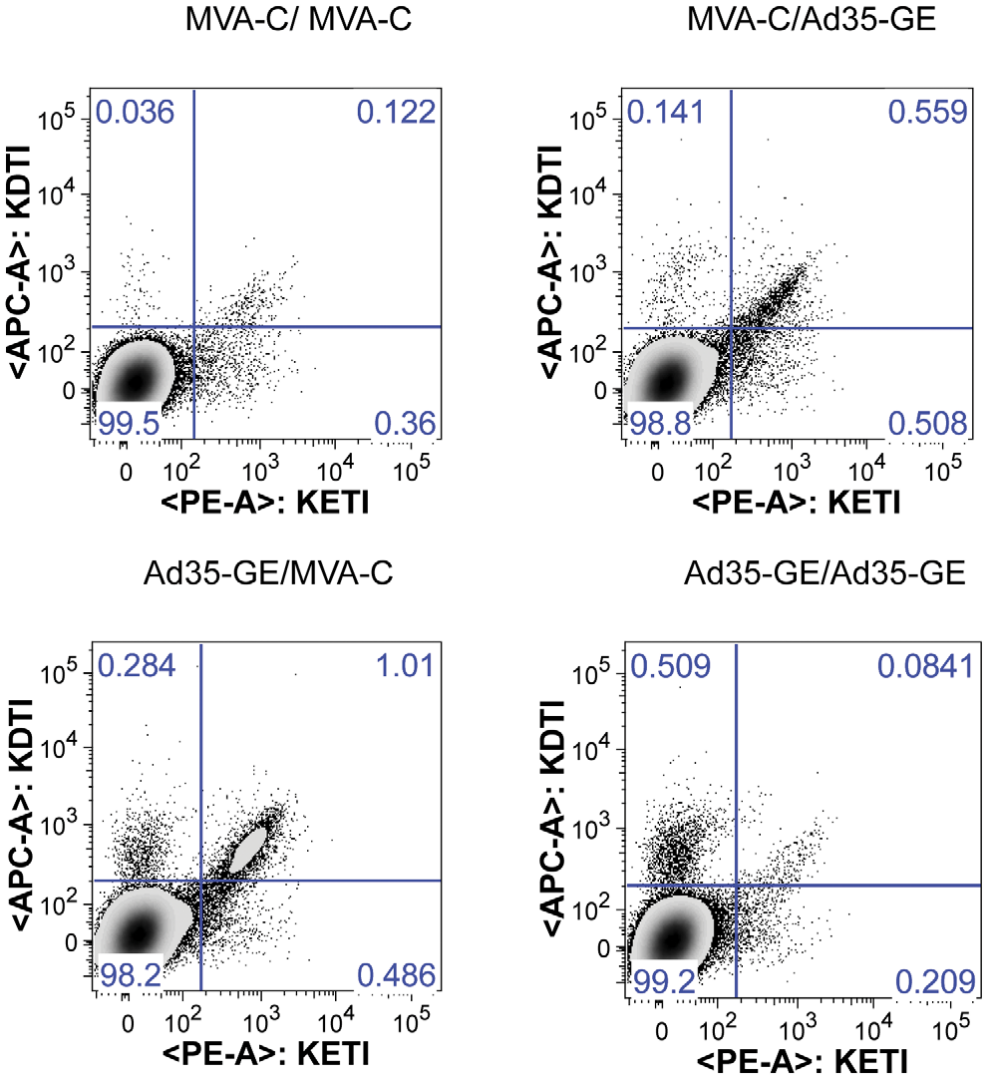
CD8+ T cells with different pentamer binding capacity are elicited by the heterologous prime-boost regimens. Depicted are the density plots of CD8+ T cell elicited by the indicated vaccine regimens and their ability to bind to the pentamer-bound Gag immunodominant motifs. The upper right quadrant represent T cell that can cross-react with both immunodominant motifs.

In an attempt to better define the kinetics and the memory phenotype of the immune response to the HIV inserts and vectors post-boost, blood was drawn from each mouse at days 24, 28, 35 and 49 (corresponding to day 3, 7, 14 and 28 post boost), pooled by group and analyzed with an 8-color flow-cytometry panel. Splenocytes were also analyzed with the same panel at days 35 and 49. At day 35, the different regimens induced T cells that could bind to both KETI and KDTI pentamers (Figure 4). In particular, the heterologous regimens were able to induce CD8+ Gag-specific T cells binding both pentamers to a greater extent compared to homologous regimens.

**Figure 5:**
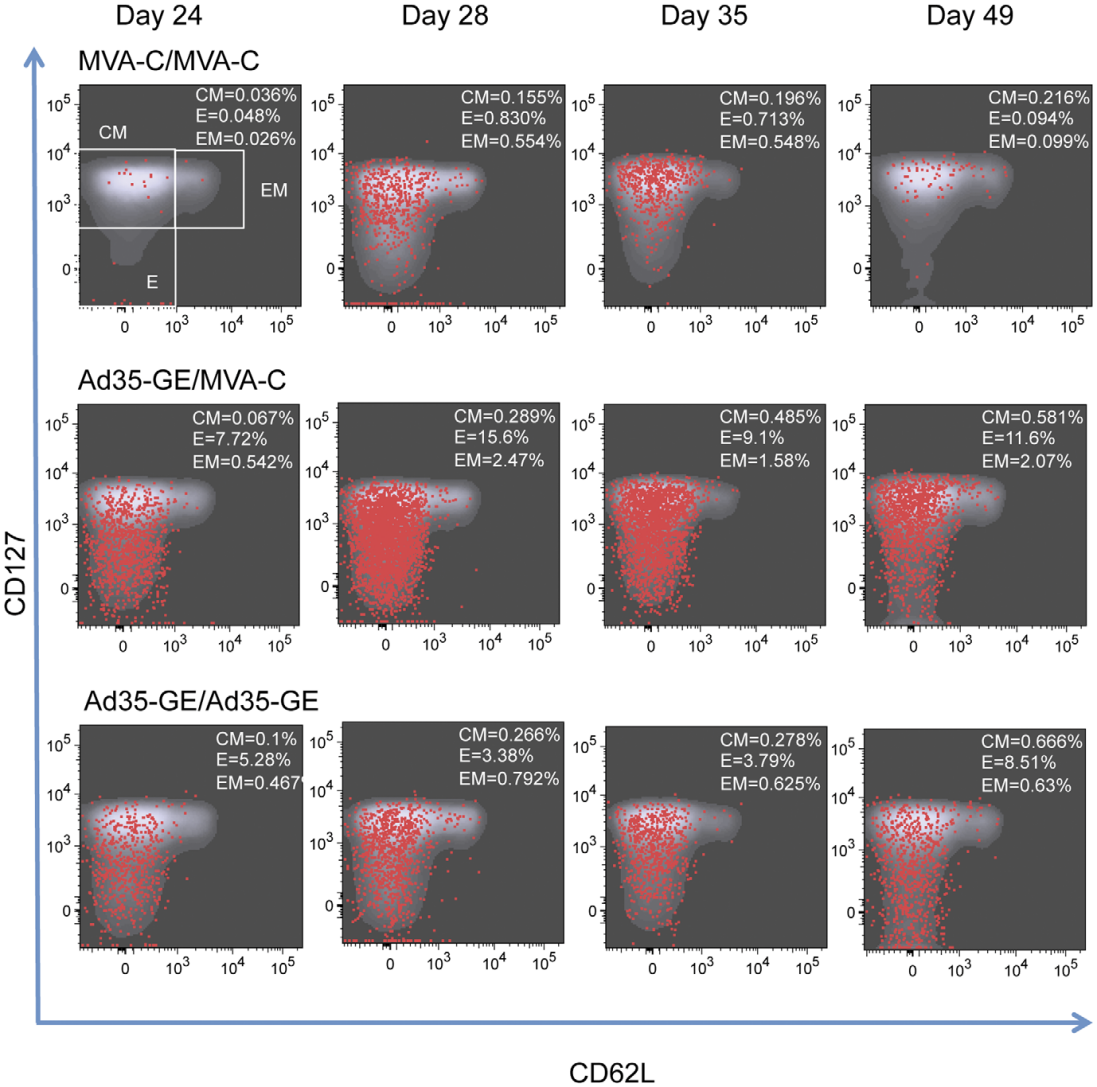
Memory phenotype of CD8+ T cells collected sequentially from mice immunized with the indicated regimens. Density plot in gray represent the memory phenotype of the cells, superimposed red dots represent the KDTI pentamer positive CD8+ T cells. Gates that define the memory subpopulations are only shown in the upper left graph, the percentage of the different memory subpopulation defined by these gates are expressed in the upper right corner in each graph. CM: central memory cells, Effector: effector cells, EM: effector memory cells.

Figure 5 shows the kinetics and dynamic expression of memory markers after homologous (top and bottom rows of graphs) or heterologous boost (middle row). Superimposed red dots represent the pentamer loaded with KDTI positive cells. The sequential graphs clearly show that the heterologous regimen induced more pentamer-specific T cells with an early predominance of two memory subsets: the effector (E) and effector memory (EM) cells. These two phenotypes transition to predominantly EM by day 35 and then contract greatly by day 49. It is interesting to note that the memory phenotypes are quite distinct in the two homologous prime-boost regimens: mice immunized with MVA-C display less effector cells at day 24, a small boost between days 24 and 28 and an overall decrease in memory cells by day 49. The mice immunized with Ad35-GE show a greater number of effector cells at day 24, which remains constant until day 49. The pentamer positive cells overlap mainly with the effector memory phenotype for MVA-C homologous regimen and the heterologous regimen (middle panel) in contrast with the effector phenotype for the Ad35-GE homologous regimen. The same analysis was performed for the KETI pentamer-specific T cells which showed a similar distribution within the memory phenotype (not shown). The peak of the response was at day 28 (corresponding to day 7 post-boost) for most of the vaccine regimens used (Figure S1)

**Figure 6:**
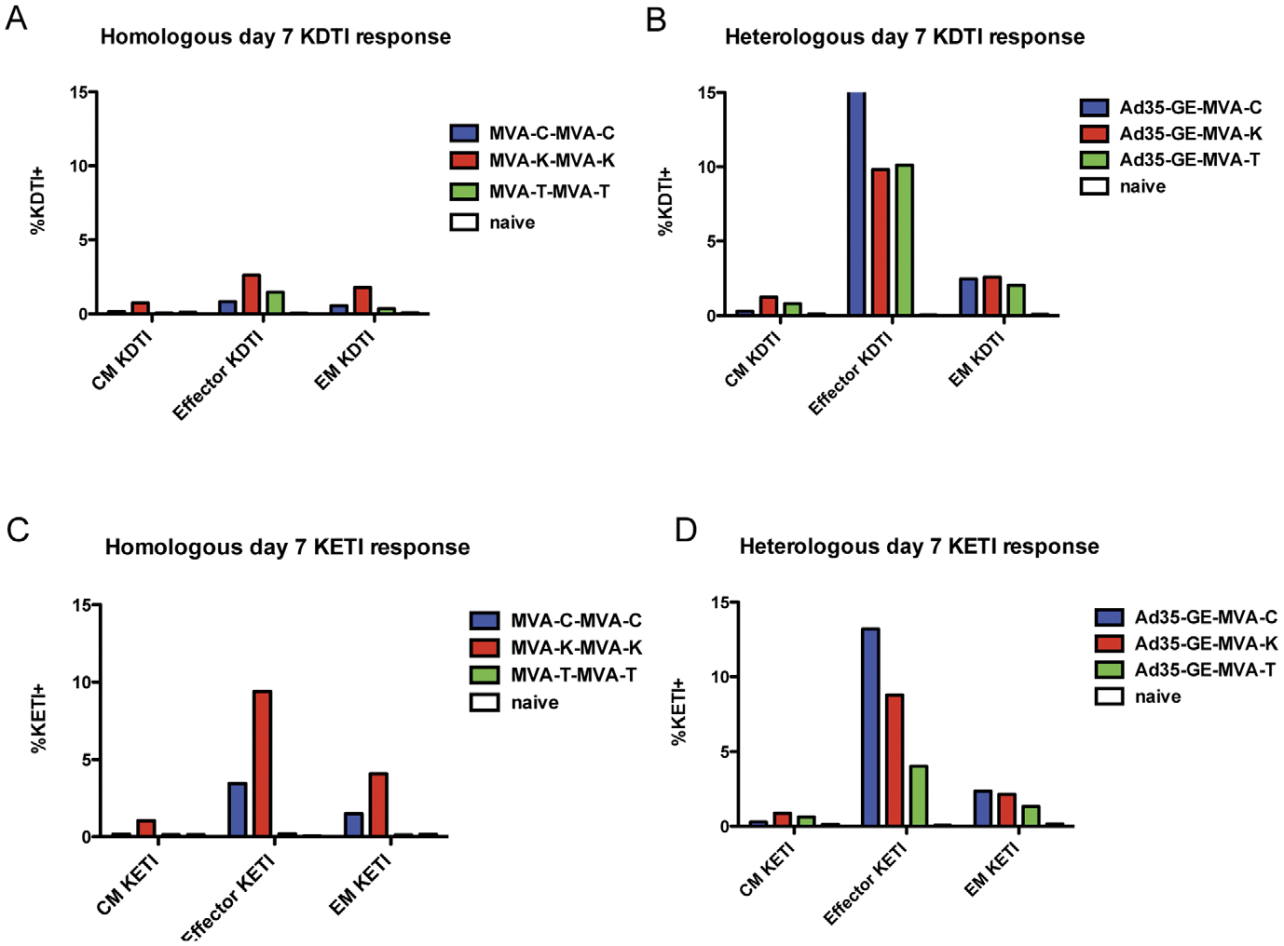
Day 7 post-boost representation of pentamer positive lymphocytes. Percentage of pentamer-binding positive lymphocytes are grouped on the x-axis according to their memory phenotype. Panels A and C show the KDTI pentamer-binding positive and panels B and D the KETI positive. Panels A and B show the phenotype of the MVA homologous regimens and panels C and D of the heterologous regimens.

When we compared the number of KDTI or KETI pentamer positive cells according to their memory phenotype at this timepoint for the other prime-boost combinations, some differences appeared (Figure 6). In particular the MVA-T homologous regimen had the lowest amount of KETI pentamer specific cells, as expected since the TZC strain does not contain the KETI sequence (Figure 6B). In contrast, the MVA-C and the MVA-K homologous regimens induced a strong response to the KDTI pentamer across the memory phenotypes even though the insert sequence for CMDR and KEA strains contains the KETI motif (Figure 6A). The heterologous regimens induced pentamer-specific CD8+ T cells in greater number and was more cross-reactive across the memory phenotypes to both motifs when compared to the homologous regimens (Figures 6C and 6D). Of note, the Ad35-GE-MVA-T regimen was able to greatly enhance the immunological responses to both immunodominat epitopes when compared to the homologous MVA-T regimen.

## Discussion

The aim of this study was to assess whether heterologous prime-boost regimens of different vaccine vectors carrying different HIV gene sequences would alter the immunodominance compared with homologous regimens and to better understand the contributions of both vectors and inserts to the phenotypic and functional profiles of the cellular immune response. The two HIV vaccines used have been tested in humans in homologous and heterologous prime-boost regimens [10], [15], [20]. Both candidate HIV vaccines induce cellular and humoral immune responses and after the boost, a strong response to the vector has been documented that overshadows the response to the HIV gene inserts (unpublished findings). Several studies have reported that heterologous prime-boost regimens induced strong immune response to the gene inserts and minimized the response to the vectors [8], [9], [11]. These reports point to a possible synergistic mechanism that is not completely understood but could lead to the development of improved vaccine combinations for future clinical trials. In an attempt to understand the mechanisms that contribute to this improved immunogenicity, we tested the Ad35-GE vaccine candidate in combination with three different rMVA vaccine constructs. The study focused on the CD8+ T-cell responses to two variants of a previously reported immunodominant epitope in Gag [21]. One was present in the Ad35-GE and MVA-T *gag* genes (AMQMLK**D**TI) and the other was present in the MVA-C and MVA-K *gag* genes (AMQMLK**E**TI).

Our data confirmed that heterologous vaccine combinations elicited consistently better CD8+ immunogenicity to the HIV gene inserts. There was a statistically significant greater response if the prime was Ad35-GE followed by rMVA constructs for the CD8+ response both in the IFN-γ ELISPOT and the ICS assays. The absence of the identical vector boost could be focusing the immune response on the insert even though the insert genes are not identical. It was also interesting that the homologous regimens focused their responses on the matched reagents while the heterologous regimens show a broader ability to recognize slightly different immunodominant epitopes. It is possible that identical boosts may select clones with highest avidity and/or specificity for the immunodominant motif while the heterologous prime-boost allows various clones that were primed with different avidity of specificity to be expanded by a slightly different motif, thus broadening the cross-recognition of the motifs. We also showed that MVA homologus regimens are weaker then homologous Ad35 regimens and that different inserts in the same MVA vector elicit different immunological responses. Table 2 shows that the Ad35-GE-MVA-T regimen where the KDTI immunodominant motif is present on both inserts elicts immune responses weaker than those induced by the heterologous vectors and heterologous inserts but are able to improve on the immunological response of the homologous MVA-T-MVA-T regimen. This observation may be partly explained by the fact that the MVA-T construct contains a less immunogenic insert sequence but that once put in an heterologous vector combination their immunogenicity is enhanced.

When pentamers loaded with each of the two immunodominant Gag motifs were used in combination, it was interesting to note that the most cross-reactive cells were induced with the heterologous regimens, while the homologous regimens selectively gave rise to CD8+ T cells that could bind to their respective insert immunodominant motif. These data imply that boosting with a different vector carrying a slightly different insert gives rise to more broadly cross-reactive T cells. Certainly, this finding supports testing heterologous inserts in HIV vaccines trials given the potential benefits of cross-reactivity considering the viral diversity of the epidemic.

Studies have shown a correlation between the presence of specific T cell memory phenotype and vaccine efficacy [22], [23], [24]. A recombinant human CMV vaccine capable of reducing post-infection SIV viral load to undetectable levels in vaccinated macaques has been shown to induce both effector and central memory T cells which might be an important association with this finding [24]. In an attempt to look at the ratio of central and effector memory T cells in this mouse model, we used pentamers loaded with the immunodominant peptide motifs KETI and KDTI in combination with memory markers and followed the kinetics of the CD8+ T cell response after boosting. The memory markers kinetics indicate that MVA-C homologous vaccination induced a slower increase of the effector cell population and a faster contraction of the same population compared with the Ad35-GE homologous vaccination or any of the heterologous regimens. This observation is strikingly similar to what has been observed in a malaria vaccine and another HIV-1 vaccine study. The Malaria study [25] reported that the persistence of central memory T cell induced by the rAd vector was protective against a malaria challenge while the HIV-1 vaccine study [26] showed that the kinetics of expansion and contraction of the memory T-cell populations are very different depending on the vector used. Similarly, our data report that the heterologous regimens at day 3 post-boost have a greater expansion of the effector and central memory population that persists to 28 days post-boost. When the pentamer specific T cells are superimposed on the density plot it is clear that the phenotype of the early response is mainly of the effector and EM phenotype, which would indicate a persistent and readily cell type population that has been described as very important in the early control of SIV infection [24]. The cross-sectional analysis of the same data offered some insight on how the insert sequence may contribute to the shaping of the response. At day 7 post-boost the mice immunized with MVA-T had the lowest binding capacity to their homologous immunodominant motif KDTI. Most of the binding resided with the effector cells implying that fully differentiated cells were the predominant cells binding to the pentamer. The other two homologous regimens gave rise after the boost to T cells that were able to bind not only their homologous peptide KETI motif but also cross-react with the KDTI peptide motif. Once the MVA-T was used in combination with Ad35-GE this ability to cross-react was enhanced but remained the lowest of the three.

In summary, our data suggest that using two different HIV-1 vaccine candidates improved the quality and magnitude of insert specific immune responses in a heterologous prime-boost regimen compared to the MVA homologous prime-boost. A recent study in rhesus macaques showed a protective effect of MVA and Ad26 heterologous vaccine regimens [27]. In addition, our data suggest that the insert sequence may play a role in shaping the quality of the immune response. This study supports the rational for continuing HIV-1 vaccine studies with combinations of heterologous vectors in a prime-boost regimen.

## Supporting Information

Figure S1 Post-boost kinetic representation of the pentamer binding. Panel A) shows the binding magnitude of CD8+ T cells to the pentamer loaded with KDTI containing peptide and panel B) the binding magnitude of CD8+ T cells to the pentamer loaded with KETI containing peptide. Graphs to the left show homologous regimens and graphs to the right the heterogolous regimens.
(TIF)
