# Supplementary figures and images for: Heterologous Prime-Boost Regimens Using rAd35 and rMVA Vectors Elicit Stronger Cellular Immune Responses to HIV Proteins Than Homologous Regimens
Source: PLoS One. 2012 Sep 26;7(9):e45840. doi: 10.1371/journal.pone.0045840 (PMC3458867; doi:10.1371/journal.pone.0045840)

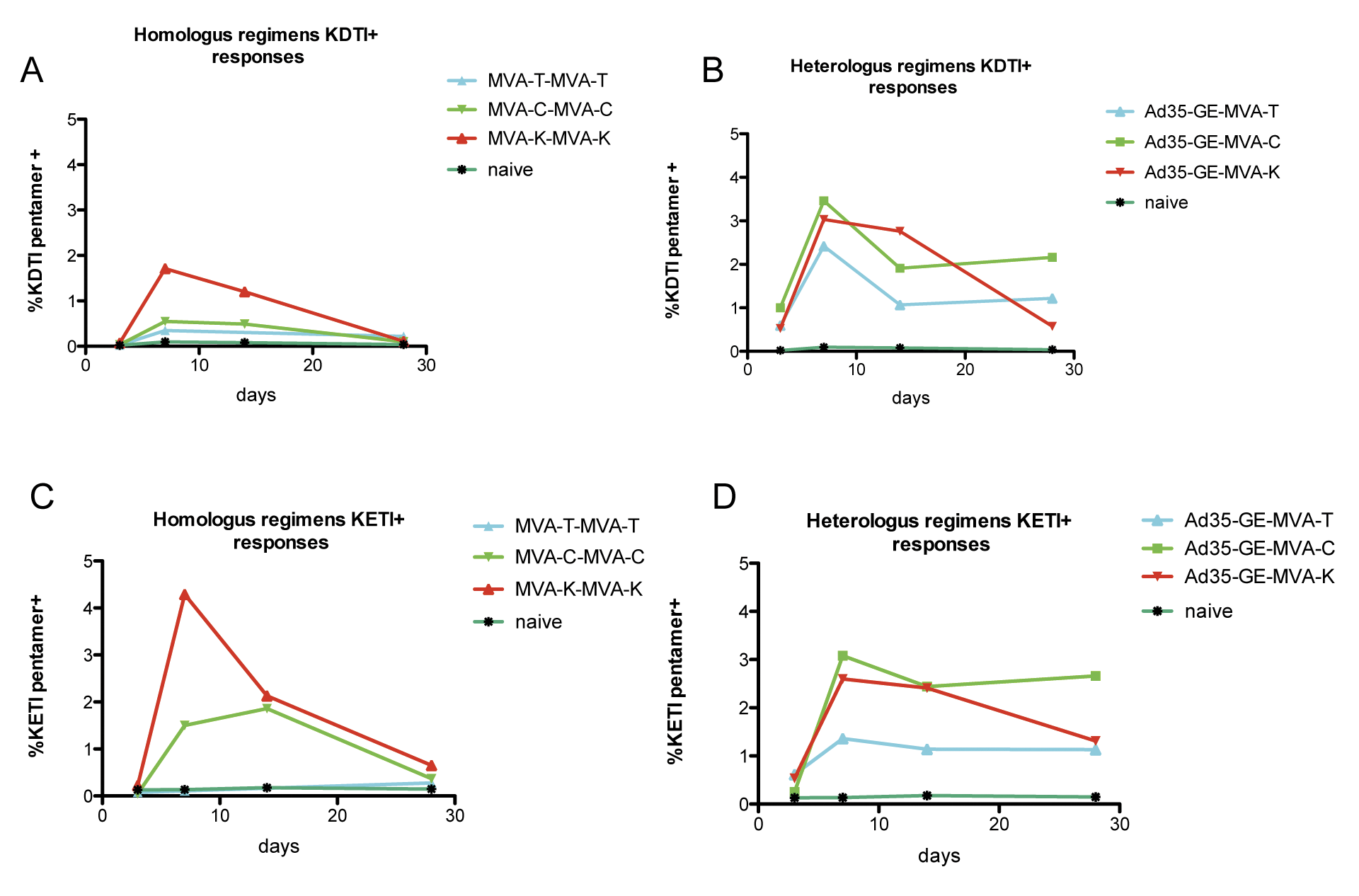

Supplement: Figure S1 — Post-boost kinetic representation of the pentamer binding. Panel A) shows the binding magnitude of CD8+ T cells to the pentamer loaded with KDTI containing peptide and panel B) the binding magnitude of CD8+ T cells to the pentamer loaded with KETI containing peptide. Graphs to the left show homologous regimens and graphs to the right the heterogolous regimens. (TIF) [file pone.0045840.s001.tif]
